# Partial molecular characterization, expression pattern and polymorphism analysis of MHC I genes in Chinese domestic goose (*Anser cygnoides*)

**DOI:** 10.1590/1678-4685-GMB-2022-0252

**Published:** 2024-07-15

**Authors:** Qianqian Zeng, Xiaojie Li, Xiaomin Shi, Shigan Yan

**Affiliations:** 1 Qilu University of Technology, School of Bioengineering, State Key Laboratory of Biobased Material and Green Papermaking, Jinan, Shandong, China

**Keywords:** Domestic goose, MHC I, polymorphism, balancing selection

## Abstract

Major histocompatibility complex (MHC) allelic polymorphism is critically important for mediating antigen presentation in vertebrates. Presently, there are insufficient studies of MHC genetic diversity in domestic Anseriform birds. In this study, we analyzed the expression profile of MHC I genes and screened for MHC I exon 2 polymorphism in one domestic goose population from China using Illumina MiSeq sequencing. The results showed that four MHC I alleles (Ancy-IE2*09/*11/*13/*21) in one goose were identified based on cDNA cloning and sequencing using four primer combinations, and the varying number of cDNA clones implied that these four classical sequences showed differential expression patterns. Through next-generation sequencing, 27 alleles were obtained from 68 geese with 3-10 putative alleles per individual, indicating at least the existence of 5 MHC I loci in the goose. The marked excess of the non-synonymous over the synonymous substitution in the peptide-binding region (PBR) along 27 alleles and five positively selected sites (PSSs) detected around the PBR indicated that balancing selection might be the major force in shaping high MHC variation in the goose. Additionally, IA alleles displaying lower polymorphism were subject to less positive selection pressure than non-IA alleles with a higher level of polymorphism.

## Introduction

The major histocompatibility complex (MHC) is an extremely variable multigene family relevant to the vertebrate immune response, encoding diverse transmembrane molecules in charge of presenting pathogenic peptides to T cells (Piertney and Oliver, 2006). MHC genes fall into at least two major groups: Class I and Class II. MHC I genes expressed in most cells are essential in the recognition and presentation of intracellular peptides (Hughes and Yeager, 1998; Sommer, 2005). MHC I genes are mainly composed of eight exons. Among them, exon 2 and 3 (abbreviated as E2 and E3) encode the α1 and α2 domains, respectively, constituting the highly polymorphic peptide-binding region (PBR) (Bjorkman and Parham, 1990).

As noted above, the MHC comprises the most diverse region in the vertebrate genome with a high degree of heterozygosity (Davies and Antczak, 1991; Zagalska-Neubauer et al., 2010; Sallaberry-Pincheira et al., 2016). Recently, MHC I genes have been widely studied among different vertebrate clades, such as marine organisms (Klein et al., 2007; Grimholt et al., 2015), mammals (Westerdahl et al., 1999; Bartocillo et al., 2021), and birds (Biedrzycka et al., 2017; Manjula et al., 2021), because of their genetic variability (Bateson et al., 2015). MHC polymorphism enables populations and individuals to produce corresponding immune responses to various pathogens and cope with changing environmental conditions (Sommer, 2005). The genetic diversity among MHC genes is affected by parasite-related balancing selection (Potts and Slev, 1995; Bateson *et al*., 2015). This balancing selection maintains genetic variation in populations based on two possible mechanisms: heterozygote advantage (Hughes and Yeager, 1998) and frequency-dependent selection (Spurgin and Richardson, 2010). Since evolving pathogens can easily escape presentation by common host alleles, rare alleles have higher fitness to survive in the host population under the prevalence of frequency-dependent selection. Hence, spatial-temporal dynamics in the pathogen community induce population variation at MHC. In the case of overdominance, individuals heterozygous in a particular gene can resist the pathogen infection better than homozygotes. In addition, sexual selection also acts to promote MHC variation (Knafler et al., 2012). 

Balancing selection was considered to be the main force for the generation and maintenance of MHC diversity (Hedrick, 1998). Nucleotide positions under positive selective pressure were expected to produce excess non-synonymous substitutions, inducing amino acid changes and structural alterations in MHC molecules (Potts and Slev, 1995; Piertney and Oliver, 2006). Such parasite-medicated selection should be notable at the PBR (Hughes and Nei, 1989; Bjorkman and Parham, 1990).

In birds, the number of MHC loci and MHC alleles variation exhibit enormous differences among different taxa. In passerines, they have a large number of MHC genes, and each locus produces a large number of alleles (Balasubramaniam et al., 2016; Pardal et al., 2017). For example, a single sedge warbler (*Acrocephalus schoenobaenus*) could carry up to 65 different alleles, indicating at least 33 MHC I loci (O’Connor et al., 2016; Biedrzycka et al., 2017). Conversely, Galliformes birds, such as chicken and turkey, has a limited number of MHC loci (Kaufman et al., 1999; Chaves et al., 2009). The chicken MHC B, called “minimal essential MHC”, has only two classical class I genes (*BF1* and *BF2*). As representatives of domestic Anseriformes bird, duck and goose, few reports on MHC I polymorphism are available (Mesa et al., 2004; Xia et al., 2004; Moon et al., 2005). Estimating population genetic variation in MHC genes is hindered by extreme MHC diversity, and the acquisition of high-coverage sequence profiles through the amplicon-throughput sequencing make it convenient and efficient to assess MHC diversity (Babik et al., 2009; Grogan et al., 2016).

The Yangzhou white goose (*Anser cygnoides, Anatidae, Anseriformes*) originated from Jiangsu Province in China, is one of the most important waterfowl breeds. Infectious diseases of domestic goose caused by pathogens, such as avian influenza viruses have broken out frequently, which severely restricts the development of the goose industry. MHC I alleles are associated with the resistance and susceptibility of pathogens (Chenani et al., 2021). Previous work published six goose MHC I full cDNA sequences and analyzed its genomic structure (Xia et al., 2005). Based on these findings, we further implement the expression profiles and the allelic polymorphism of MHC I E2 of one domestic goose population. The major goals in this study are to (i) characterize the genetic polymorphism of MHC I E2 alleles through Illumina MiSeq, (ii) determine the role of positive selection and recombination in shaping class I variation, and (iii) infer the evolutionary relationship of Anseriformes.

## Material and Methods

### Sample preparation

Sixty-eight blood samples of adult Yangzhou white geese (hereinafter referred to as YW-goose or goose) were collected by brachial venipuncture for DNA extraction. The animal studies were performed under the approval of the Animal Ethics Committee of Qilu University of Technology (Shandong Academy of Sciences). Genomic DNA was extracted by a standard phenol-chloroform protocol (Sambrook et al., 1990). Additionally, the blood and three kinds of fresh organs (heart, lung, and intestine) from one YW-goose were preserved in liquid nitrogen as an mRNA resource. Total RNA was extracted by Trizol reagent (Vazyme Biotech Co. Ltd). The first strand cDNA was synthesized by reverse transcription according to the instruction of the HiScriptⅢRT SuperMix for qPCR kit (Vazyme Biotech Co. Ltd). cDNA samples were stored at -80 °C.

### Primer design, molecular cloning and Sanger sequencing

The first set of new primers (E2-4IF/E2-4IR; Table S1) amplified approximately 1697 bp spanning from E2 to partial E4, on the basis of an alignment comprising MHC I sequences from domestic goose (AY387652; AY387658; AY387651; AY387650; AY387648; AY387699), domestic chicken (M31012), bar-headed goose (FJ606105; FJ606106; FJ606107; FJ606108; FJ606109) and duck (AB115244; AY294419). Then, three other primer pairs (E2AF/E2AR, E2IF/E2IR, E3I20F/E3I30R; Table S1) were designed to amplify E2 and E3, respectively. Both gDNA and cDNA samples were used as PCR templates. The 25 µL PCR reaction system contains the following components:5 µl of PCR enhancer, 12.5 µL of 2×phanta max master Mix, 20 ng of blood DNA template, 2 µM of each primer , add water to a total volume of 25 µL. The PCR program consisted of one cycle at 95 °C for 5 min, followed by 36 cycles of amplification at 95 °C for 15 s, Tm for 15 s and 72 °C for 0.5-1.5 min, and a final amplification at 72 °C for 5 min. The PCR products of the correct length were cloned and then the randomly-selected positive clones were sequenced using M13 primers. The combined sequences from different primer pairs gave satisfactory coverage of the hypervariable region E2 and E3, which encoded the peptide-binding region (Pardal et al., 2017). 

### Illumina MiSeq sequencing and data analysis

According to the above sequence alignments obtained through Sanger sequencing, the universal primer pair E2AF/E2AR (Table S1) was newly devised for the MiSeq sequencing, which amplified a 176bp fragment (excluding the primer sequences) of MHC I E2. After being purified by the KAPA Pure Beads (Roche), each PCR product labelled with a unique barcode was sequenced using a 2×250 bp paired-end sequencing strategy on the Illumina NovaSeq 6000 system. Raw FASTQ reads were demultiplexed using the barcode sequence with the exact barcode matching parameter (https://github.com/jameslz/div-utils/blob/master/div-utils, version: 0.0.1-r1-dirty). Quality-filtering utilized by the Trimmomatic version was performed as in Bolger et al. (2014). Paired reads were merged using the USEARCH command (http://drive5.com, version 11.2.64) (Edgar and Flyvbjerg, 2015) with the default parameters. Reads that cannot be merged were discarded; the merged reads with more than two nucleotide mismatches in primer matching and the primer sequences from the merged reads were deleted.

### Validation and identification of MHC I alleles

All alleles from cloning and Illumina sequencing were verified in BLASTN (NCBI; National Council for the Blind of Ireland) to confirm whether they were MHC I alleles. We performed repeated PCR verification for most of the suspicious alleles to exclude PCR artifacts. When a clone was found in more than two separate PCRs or an allele obtained by Illumina sequencing was found in more than two samples, it was considered an MHC I allele that met the criteria summarized by Kennedy et al. (2002). According to the nomenclature of Klein et al. (1990), we simply named our alleles Ancy-IE2*01-27. Unique alleles were uploaded to the GenBank database and specific codes were obtained (GenBank accession number: OK289674-OK289700). Specifically, the 10 E2 alleles (Ancy-IE2*01-10) shared the conserved sequence region, hence the specific primers (E2IF/E2QIR) were designed to determine whether there truly existed such an MHC I locus (hereinafter called as IA.I stands for MHC class I, and A refers to the locus ordinal number) in the goose.

### Analysis of allelic diversity, positive selection and recombination

We analyzed MHC I E2 sequences in MEGA X (Kumar et al., 2018). The diversity analysis included the average nucleotide distance (*d*_*nt*_ ), average amino acid distance (*d*_*aa*_ ), and average nucleotide diversity (*π*). The *d*_*nt*_ and *d*_*aa*_ adopted the Kimura 2-parameter model (Kimura, 1980) and P-distance model, respectively, and both tests were run with 1000 bootstrap repeats. 

For the selection effect of MHC I gene, firstly, we used the modified Nei-Gojobori method (Nei and Gojobori, 1986) in MEGA X to calculate the average rate of non-synonymous (*d*_*N*_ ) and synonymous (*d*_*S*_ ) substitution for all codons, the PBR and non-PBR region. Fourteen PBR codons (1V, 10V, 19M, 38D, 41S, 42S, 44S, 45N, 48I, 49Y, 51V, 52N, 55T, 56L) were verified among goose MHC I E2 alleles based on human and chicken MHC I molecules (Kaufman et al., 1992; Wallny et al., 2006). Secondly, we applied two software to detect the specific positive selection sites (PSSs). The maximum likelihood (ML) as a random site mode, could better identify the PSSs since it described the overall change among sites (Furlong and Yang 2008). The nested site models [M7 (0 <ω<1) and M8 (ω>1)] in PAML (Yang, 2007), which assumed that all sites presented a beta distribution, were used for the comparison to determine PSSs. Two models, FEL (Fixed effects likelihood) and MEME (Mixed effects model of evolution), were performed through the Data-monkey web server (http://www.datamonkey.org/).

To test the recombination events occurring at MHC I genes of the goose, the genetic algorithm of the RDP (GARD) was also performed in the Data-monkey web server.

### Phylogenetic relationship

In order to evaluate the phylogenetic relationship of MHC I genes among the Anseriform species, all the MHC I E2 alleles of goose identified in this article and the corresponding sequences of six other Anseriform species from GenBank were used for phylogenetic reconstruction. The human MHC I sequence (K02883) was used as an outgroup. Phylogenetic trees were constructed by the neighbor-joining (NJ) method in MEGA X with 1000 rapid bootstrap replicates. Nucleotide-based trees was built using Tamura-Kumar models (Tamura, 1992). The network was built by Splits Tree v.4.14.4 based on the substitution model the Kimura 2-parameter (K2P) with 1000 bootstrap repetitions.

## Results

### cDNA analysis of goose MHC I genes

**Figure 1 -:**
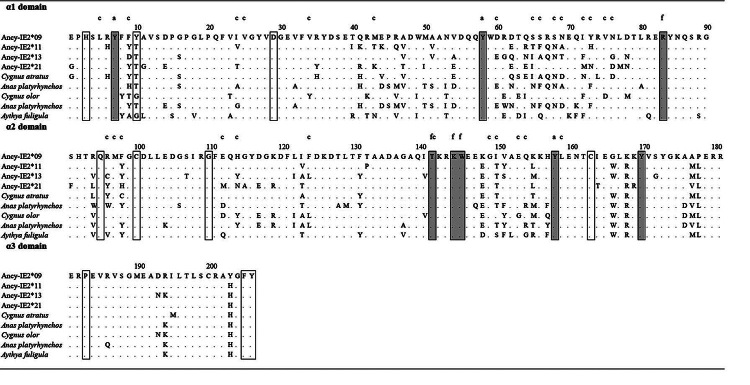
Amino acid alignment of MHC I long transcripts in the domestic goose. The same sites as the Ancy-IE2*09 alleles were indicated by “·”; the transparent boxes represented the inter-domain and intra-domain contact residues and the light gray boxes represented the peptide main chain sites; c represented non-main chain peptide contacts, while a and f represented A and F pocket, respectively. The Anseriform sequences from NCBI included *Cygnus atratus* (XR004782210.1); *Anas platyrhynchos* (GU245865.1); *Cygnus olor* (XM040542813.1); *Anas platyrhynchos* (KX118687.1); *Aythya fuligula* (XM032205162.1).

Sanger sequencing using four pairs of primers from both gDNA and cDNA of one single YW-goose confirmed MHC I sequences of different lengths (201bp-1697bp; Table S1), identifying a total of four different alleles (Ancy-IE2*09, Ancy-IE2*11, Ancy-IE2*13, and Ancy-IE2*21; Figure 1). 109 cDNA clones were obtained from the same individual. Among them, Ancy-IE2*13 and Ancy-IE2*21 were in the vast majority, accounting for 44% and 39.4% respectively, while Ancy-IE2*09 and Ancy-IE2*11 only accounted for 7.3% and 9.2%, respectively (Table S2). These four long transcripts covered the major part of E2-4: α1 (90 amino acids), α2 (90 amino acids), and partial α3 (70 out of 92 amino acids) domain. In non-mammalian vertebrates, the classical MHC I amino acid sequences comprised eight highly conserved sites “YYRTKWYY” (Kaufman et al., 1994; Shum et al., 1999), which were termed as peptide main-chain sites, and similarly, these eight sites were all embodied in the MHC I transcripts of goose and four other Anseriform species (*Cygnus atratus; Cygnus olor; Anas platyrhynchos; Aythya fuligula*). Besides, classical MHC molecules were extremely conservative in inter- and intra- domain contact residues (Shum *et al*., 1999). In the Anseriformes transcripts, 10 out of the 18 inter- and intra- domain contact residues were included: 9 positions remained unchanged, and only one position was highly variable in the alignment (Figure 1). cDNA sequence and expression analysis collectively illustrated that at least four classical alleles with distinct expression patterns existed in this single YW-goose individual. 

### Exon 2 sequences from Illumina MiSeq

383 MHC I sequences (176 bp) in 68 individuals were detected using next-generation sequencing, and eventually, a total of 27 MHC I alleles were determined after sequence alignment (Table S3). 27 MHC I alleles from Illumina MiSeq were translated into 21 unique amino acid sequences, which covered most of the PBR of the α1 domain (14/18). The number of alleles for each individual varied greatly from 3 to 10. Thus, it was speculated that there were at least 5 MHC I loci in the goose. Six alleles were present in more than half of the individuals (Ancy-IE2*01(50/68), Ancy-IE2*09(45/68), Ancy-IE2*11(47/68), Ancy-IE2*13(48/68), Ancy-IE2*014(37/68), Ancy-IE2*16 (56/68)), while fifteen alleles only appeared in 2-7 individuals (Table S3).

Among the 176 nucleotide positions of the 27 MHC I alleles, 54% were variable. There was no premature stop codon in the putative amino acid sequences, implying that these alleles might be functional. The average nucleotide diversity (*π*), nucleotide distance (*dnt*) and amino acid distance (*daa*) of the PBR region in goose MHC I E2 were significantly higher than the non-PBR region (Table 1).

**Table 1 -:** Measures of average nucleotide diversity (*π*) and nucleotide distance (*dnt*) and amino acid distance (*daa*) for goose MHC I exon 2 alleles.

	π	dnt±SE	daa±SE
All	0.126	0.143±0.018	0.214±0.032
All PBR	0.229	0.297±0.063	0.381±0.77
All non-PBR	0.094	0.104±0.060	0.161±0.031
Loci IA	0.04	0.044±0.008	0.064±0.015
Loci IA PBR	0.0047	0.005±0.005	0.014±0.014
Loci IA non-PBR	0.022	0.023±0.008	0.032±0.018
Loci non-IA	0.131	0.150±0.018	0.362±0.078
Loci non-IA PBR	0.21	0.262±0.060	0.362±0.079
Loci non-IA non-PBR	0.113	0.128±0.022	0.211±0.037

### Positive selection and recombination of goose MHC I exon 2 alleles

Calculations of *d*_*N*_ (non-synonymous substitution) and *d*_*S*_ (synonymous substitution) were listed in Table 2. All MHC E2 alleles yielded higher *d*_*N*_ than *d*_*S*_ values in all codons, PBR and non-PBR; moreover, the *d*_*N*_ /ds of PBR was higher than that of non-PBR (2.08, 1.657, respectively), although its *d*_*N*_ and *ds* values were slightly less than that of non-PBR codons.

**Table 2 -:** Calculations of non-synonymous (*dN*) and synonymous (*dS*) substitutions for the goose MHC I exon 2 alleles.

	d_*N*_±SE	d_*S*_±SE	d_*N*_/d_*S*_
All	14.26±1.28	7.812±1.407	1.825
All PBR	6.499±1.339	3.125±0.764	2.08
All non-PBR	7.766±1.487	4.687±1.112	1.657
Loci IA	3.711±0.857	3.4±0.867	1.091
Loci IA PBR	0.200±0.186	0	-
Loci IA non-PBR	1.156±0.6	1.126±0.595	0.912
Loci non-IA	15.904±2.377	7.074±1.371	2.248
Loci non-IA PBR	6.441±1.459	2.404±0.768	2.679
Loci non-IA non-PBR	8.430±1.609	3.820±0.919	2.206

“-” stands for infinity.

Five PSSs within non-IA alleles were detected using three methods based on the detection of PSSs for 58 codons of E2, while only one PSS within IA alleles (Table S4). Five PSSs were identified through the likelihood ratio test of comparing the nested models M7 and M8. Two PSSs were detected in FEL and MEME. However, there were few sites within E2 under positive selection using four methods, all the PSSs located in or around PBR, which were directly related to peptide binding.

Recombination studies were also carried out using GARD among 27 alleles; the results showed that no recombination point was found, mainly because of the short sequence length.

### Locus determination and polymorphism analysis of IA

**Figure 2 -:**
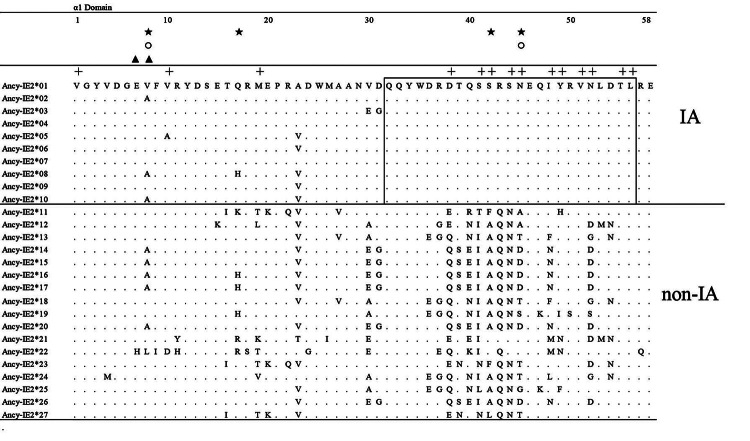
Amino acid alignment of 27 MHC I exon 2 alleles of domestic goose. “+” indicated the predicted PBR site. “★” indicated the positively selected sites (PSSs) predicted by M7 and M8 site model, while “○” and “▲” were the PSSs detected by FEL and MEME models, respectively. The locus-specific residues of IA alleles were indicated by open boxes.

By aligning 27 amino acid sequences, it was impossible to assign all the sequences to separate loci by visual inspection. However, it was found that the specific amino acid motif composed of multiple conserved amino acid residues in an α helix and its vicinity was shared by ten alleles **(**Ancy-IE2*01-10, Figure 2), resembling that of chicken *BF1* (Livant et al., 2004), golden pheasant *IA1* (Zeng et al., 2016), and human *HLA-C* (Zemmour and Parham, 1992) alleles, which could be used to distinguish individual loci. Based on this remarkable sequence feature, we preliminarily determined that these ten sequences belonged to one gene locus-locus IA. For the IA locus, no more than two alleles were identified per YW-goose by sequence-specific PCR and Sanger sequencing, further elucidating that such a single locus truly existed.

The polymorphisms of ten alleles of predicted locus IA were analyzed (Table 1), which had few variant sites, and the amino acid diversity, nucleotide distance and amino acid distance were extremely low, indicating that the locus IA had relatively low genetic diversity. Since they were highly conserved around the PBR, the *π*, *d*_*nt*_ and *d*_*aa*_ values of the PBR region were lower than other codons. Nevertheless, a higher *d*_*N*_ than *d*_*S*_ value was observed in all codons and PBR (Table 2), suggesting locus IA might be subjected to positive selection.

### Phylogenetic analysis

**Figure 3 -:**
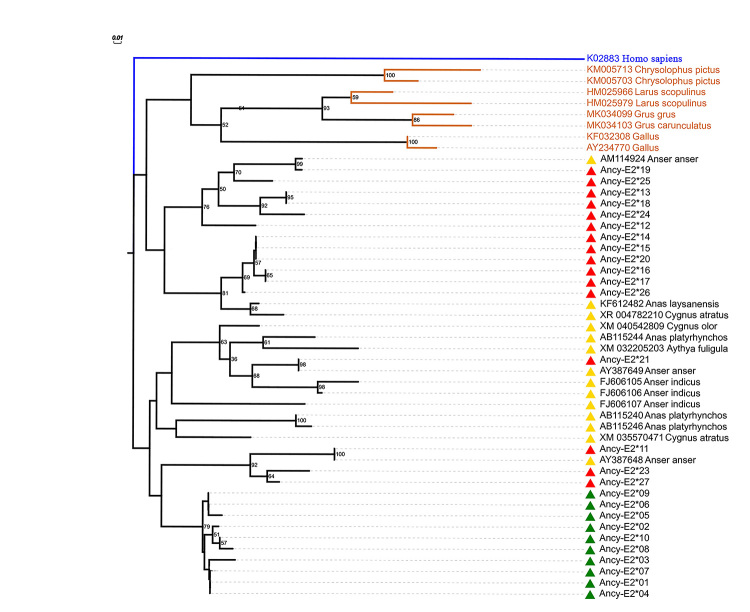
Phylogenetic tree of nucleic acid (a) and amino acid (b) sequences for domestic goose MHC I exon 2. The sequences used to generate the trees were as follows: *Chrysolophus pictus* (KM005713, KM005703); *Larus scopulinus* (HM025966, HM025979); *Grus grus* (MK034099); *Grus carunculatus* (MK034103); *Gallus* (KF032308, AY234770); *Anser anser* (AM114924, AY387649, AY387648); *Anser indicus* (FJ606105, FJ606106, FJ606107); *Cygnus olor* (XM040542809); *Anas platyrhynchos* (AB115240, AB115244, AB115246); *Cygnus atratus* (XR004782210, XM035570471); *Aythya fuligula* (XM032205203); *Anas laysanensis* (KF612482); *Homo sapiens* (KO2883). Only bootstrap values >50% were shown at the node. The red triangle represented non-IA alleles of domestic geese, the green triangle represented IA alleles of domestic geese, and the yellow triangle represented class I sequences from other Anseriform species. *Homo sapiens* (KO2883) as the outgroup was shown in blue and sequences from non-Anseriform bird were shown in red.

The goose MHC I sequences clustered with the previously reported MHC I sequences from other Anseriformes species, such as *Anser anser*, *Anser indicus, Cygnus olor*, *Anas platyrhynchos, Cygnus atratus, Aythya fuligula and Anas laysanensis*. At the same time, they diverged from those of non- Anseriformes birds both in the nucleic acid (Figure 3) phylogenetic trees. Within the Anseriformes clade, sequences from different species were inclined to gather together. For the IA locus alleles of geese, all alleles formed an independent cluster with a supporting rate of more than 75%, indicative of the independent evolution of this locus; for non-IA alleles, mostly fell into two major clusters due to sequence divergence while some mingled with MHC I sequences from other Anseriform species (Ancy-IE2*11/21/23/27). Besides, the network relationship among MHC I sequences of Anseriform species resembled the situation described above but was not shown here.

## Discussion

### The determination of goose classical MHC I sequences

In this study, we isolated four MHC I alleles from the cDNA of a single YW-goose, spanning the region from E2 to partial E4. Four alleles that could express were detected by different primer combinations and displayed sequence characteristics of the classical MHC I genes, i.e., conserved peptide main-chain sites and inter- and intra- domain contact residues (Kaufman et al., 1994). Previous studies showed that the relative cDNA clone number of each sequence was a good indicator of the expression levels of these genes (Mesa et al., 2004). Hence, through the number of cDNA clones of the four alleles, we speculated that the expression levels of the four alleles might be different: two highly expressed alleles (Ancy-IE2*13 and Ancy-IE2*21) and two relatively weakly expressed alleles (Ancy-IE2*09 and Ancy-IE2*11). The differential expression pattern between different MHC classical loci implied differences in their capacity for defense against viruses, which was commonly found in other species (Zemmour and Parham, 1992; Biedrzycka et al., 2017): *BF2* was dominantly expressed compared with *BF1* in the chicken (Kaufman, 2020).

### Extensive MHC allelic variation in the YW-goose population

It was the first time we characterized the E2 of MHC I genes in one population of YW-geese using the Illumina MiSeq. We founded that each individual contained 3-10 alleles, indicating that there were at least 5 class I loci in the goose. Similarly, the MHC of the Duck, as a closely related species of goose, reportedly contained 5 class I loci (*UAA*, *UBA*, *UCA*, *UDA*, and *UEA*) (Moon et al., 2005). In total, 27 distinct MHC I E2 alleles were identified from 68 individuals, revealing a high genetic variation of ɑ1 domain in the goose class I genes. Other avian species where they also reported to exhibit the extensive polymorphism at the peptide-binding domains of MHC I genes (Pardal et al., 2017). Especially Passerine species showed extreme polymorphism at MHC genes, e.g., 88 MHC I alleles obtained from 18 siskins (*Spinus spinus*) (Drews and Westerdahl, 2019). The abundant sequence variation of YW-goose MHC I loci might enable this species to recognize and present a large number of pathogenic antigens (Potts and Slev, 1995). 

According to the allele distribution of 68 individuals, Ancy-IE2*01 (50/68) and Ancy-IE2*09 (56/68) were the most common alleles, indicating that these two alleles might be favoured by selection in this YW-goose population (Oliver et al., 2009). There were also 14 alleles only appeared in 2-3 individuals, suggesting many rare alleles had evolved in this population under frequency-dependent selection, the fundamental process contributing to the generation and maintenance of MHC variability (Hughes and Nei, 1989; Takahata and Nei, 1990). Furthermore, five alleles (Ancy-IE2*01, Ancy-IE2*09, Ancy-IE2*11, Ancy-IE2*13, Ancy-IE2*19) were also detected in the geese from Beijing (Xia et al., 2005), indicating that these alleles were shared among different areas. This situation implied that two different populations of domestic geese experienced very similar pathogen-mediated selective regimes, which was commonly reported in birds, such as owls (Burri et al., 2008), penguins (Sallaberry-Pincheira et al., 2016), and passerines (Eimes et al., 2016).The discovery of these alleles laid the foundation for the breeding and disease resistance research of goose.

### Locus identification of IA

Previous studies verified that E2 sequences from domestic chicken *BF1* (Piertney and Oliver, 2006), golden pheasant *IA1* (Zeng et al., 2016), and human *HLA-C* (Zemmour and Parham, 1992) shared a locus-specific amino acid motif, and such a phenomenon also appeared among ten MHC I alleles of the goose (Ancy-IE2*01-10). Based on this striking finding, these ten alleles most likely have classified into the IA locus of the goose. Besides, the locus-specific PCR amplification results proved the existence of the IA locus. Undoubtedly, the determination of locus IA in geese would contribute to further elucidation of the evolutionary processes underlying MHC variation at such a particular locus. 

### Differential polymorphism caused by different selection pressure

Based on the defined criteria, *d*_*N*_ /*d*_*S*_ >1 was usually considered as evidence of positive selection for MHC diversification. For all the 27 E2 alleles of goose MHC I, positive selection exerted an influence on not only PBR codons but also non-PBR codons, which both exhibited an obvious excess of non-synonymous over synonymous substitutions. Furthermore, E2 alleles showed a higher *d*_*N*_ /*d*_*S*_ ratio at PBR codons than that at non-PBR codons, which conformed to the hallmark of functionally important residues under balancing selection. However, compared with 17 non-IA alleles, ten alleles of the IA locus exhibited less allele polymorphisms and fewer frequent nucleotide mutations, implying less selection pressure acting on the functional variation at IA. Previous studies demonstrated that the difference of allele diversity at MHC genes would result from the difference of selection intensities acting on different loci. In humans, *HLA-A* and *HLA-B* was more polymorphic due to stronger selection pressure than *HLA-C* loci (Zemmour and Parham, 1992). In the alpine newt, the *DAB* gene produced more mutations due to strong positive selection, while *DBB* gene produced only a small number of mutations due to lack of selection pressure (Babik et al., 2008). 

According to the predictive results of 4 codon-based models, we got some evidence of balancing evidence acting on specific codons, as well as divergent selective pressure between IA alleles and non-IA alleles. Five PSSs were determined in non-IA alleles; all fell into the PBR and its vicinity, which was consistent with the signs of functionally important areas as targets for balancing selection (Zhang et al., 1998). A larger substitution happened at those specific codons of non-synonymous than synonymous under positive selection (Figure 2), demonstrating that these variations within the α1 domains might be functionally important (Kuduk et al., 2012). Contrarily, fewer PSSs were detected at IA. Therefore, selective pressure on IA alleles and non-IA alleles differed strikingly, and IA locus was less affected by selection pressure. In conclusion, differing levels of diversity and differential selection might indicate different functional roles for the goose loci. Moreover, although we did not detect recombination events in E2 alleles, we could not completely exclude the possibilities of inter-locus recombination.

### The phylogenetic relationship in Anseriformes

In order to evaluate the goose phylogenetic relationship, we constructed a phylogenetic tree including domestic geese and some other birds. In Anseriformes, goose alleles did not cluster together, but scattered on the tree with other species of Anseriformes. The higher similarity of sequences between rather than within species (trans-species similarity) was usually explained with trans-species polymorphism (TSP). Under the effect of balanced selection and others, TSP was the maintenance and sharing of favorable functionally important alleles of immune-related genes between species. Although trans-species similarity could be also explained with convergent evolution, most avian MHC studies indicated that balanced TSP was a predominant mechanism responsible for trans-species alleles (Sallaberry-Pincheira et al., 2016), such as Accipitriform (Minias et al., 2019), Passerines (Chen et al., 2015; Balasubramaniam et al., 2016; Gillingham et al., 2016) and Spheniscidae (Kuduk et al., 2012).

## Conclusions

In this study, we first examined the tissue expression levels of MHC I genes in one goose, conducing that in this goose there existed at least four classical sequences displaying differential expression patterns. It was the first time that we genotyped E2 of MHC I genes of 68 individuals from one YW-goose population by Illumina MiSeq sequencing, revealing that: 1) balancing selection might be the main force to shape the high MHC allelic variation in this goose population; 2) IA alleles and non-IA alleles exhibited differential genetic polymorphisms, and weaker positive selection detected at IA than non-IA alleles might account for much less variation of IA than non-IA alleles; 3) YW-goose alleles might have a trans-species polymorphism in Anseriformes.

## Supplementary material

The following online material is available for this article:

Table S1 - Primers used to amplify MHC I sequences in the domestic goose

Table S2 - Numbers of cDNA clones amplified by four pairs of primers

Table S3 - Statistics of MHC I exon 2 alleles from 68 domestic geese

Table S4 - Inference of positively selected amino acid sites for domestic goose MHC I sequences
