# Supplementary material for: Partial molecular characterization, expression pattern and polymorphism analysis of MHC I genes in Chinese domestic goose (Anser cygnoides)
Source: Genet Mol Biol. 2024 Jul 15;47(2):e20220252. doi: 10.1590/1678-4685-GMB-2022-0252 (PMC11249561; doi:10.1590/1678-4685-GMB-2022-0252)
Supplement: Table S1 - [file 1415-4757-GMB-47-02-e20220252-s1.pdf]

Genetics and Molecular Biology

DOI: https://doi.org/10.1590/1678-4685-GMB-2022-0252

Supplementary Material to "Partial molecular characterization, expression pattern and polymorphism analysis of MHC I genes in Chinese domestic goose (*Anser cygnoides*)"

Table S1 - Primers used to amplify MHC I sequences in the domestic goose.

	**Primer name**	**Primer sequence (5'- 3')**	**Coverage**	**Sequence length (bp)**	**Tm (°C)**
1	E2AF	KCCGCAGTTCGTGRTY	PE2	210	54
	E2AR	CCCCTGCTCTGGTTGTAG			
2	E2IF	GCCCCACTCCCTGCRYTATTTC	E2	262	52
	E2IR	CCCTGCTCTGGTTGTRG			
3	E2-4IF	GGAGCAGCGAGCGGTGA	E2＋E3＋PE4	1697/781	60
	E2-4IR	GCGGCACTGGTACTTGTCC			
4	E3I20F	ATGTWTGGCTGTGACCTCCT	PE3	201	52
	E3I30R	CRCATATTTCYTCAGCCMCTC			
5	E2IF	GCCCCACTCCCTGCRYTATTTC	PE2	253	54
	E2QIR	GCAGTGTGTCCAGATTCACACGAT			

Tm represents the annealing temperature. PE is the abbreviation for the partial exon and E is short for the entire exon in the vertical column of Coverage. "1697" and "781" represents the length of the gDNA and cDNA sequence, respectively.
