# Supplementary material for: Partial molecular characterization, expression pattern and polymorphism analysis of MHC I genes in Chinese domestic goose (Anser cygnoides)
Source: Genet Mol Biol. 2024 Jul 15;47(2):e20220252. doi: 10.1590/1678-4685-GMB-2022-0252 (PMC11249561; doi:10.1590/1678-4685-GMB-2022-0252)
Supplement: Table S2 - [file 1415-4757-GMB-47-02-e20220252-s2.pdf]

Genetics and Molecular Biology

DOI: https://doi.org/10.1590/1678-4685-GMB-2022-0252

Supplementary Material to "Partial molecular characterization, expression pattern and polymorphism analysis of MHC I genes in Chinese domestic goose (*Anser cygnoides*)"

Table S2 - Numbers of cDNA clones amplified by four pairs of primers.

Allele	E2-4IF/E2-4IR	E2IF/E2IR	E2AF/E2AR	E3I20F/E3I30R	Total (clone percentage)
Ancy-IE2*09	3	1	2	2	8（7.3 %）
Ancy-IE2*11	2	3	3	2	10（9.2 %）
Ancy-IE2*13	9	14	12	13	48（44.0 %）
Ancy-IE2*21	10	12	10	11	43（39.4 %）
Total	24	30	27	28	109

The four cDNA sequences from one goose refer to Ancy-IE2*09, Ancy-IE2*11, Ancy-IE2*13 and Ancy-IE2*21. These primer combinations for cDNA amplification refer to E2-4IF/E2-4IR, E2IF/E2IR, E2AF/E2AR and E3I20F/E3I30R.
