# Supplementary material for: Partial molecular characterization, expression pattern and polymorphism analysis of MHC I genes in Chinese domestic goose (Anser cygnoides)
Source: Genet Mol Biol. 2024 Jul 15;47(2):e20220252. doi: 10.1590/1678-4685-GMB-2022-0252 (PMC11249561; doi:10.1590/1678-4685-GMB-2022-0252)
Supplement: Table S3 - [file 1415-4757-GMB-47-02-e20220252-s3.pdf]

Genetics and Molecular Biology

DOI: https://doi.org/10.1590/1678-4685-GMB-2022-0252

Supplementary Material to "Partial molecular characterization, expression pattern and polymorphism analysis of MHC I genes in Chinese domestic goose (*Anser cygnoides*)"

Table S3 - Statistics of MHC I exon 2 alleles from 68 domestic geese.

**Sample**		**IA**											**non-IA**																	
**Number**	**Numer of all alleles**	*01	*02	*03	*04	*05	*06	*07	*08	*09	*10	**Number of IA alleles**	*11	*12	*13	*14	*15	*16	*17	*18	*19	*20	*21	*22	*23	*24	*25	*26	*27	**Number of non-IA alleles**
DG01	5		√						√			2			√		√	√												3
DG02	6	√	√									2	√		√	√		√												4
DG03	6	√	√									2	√		√	√		√												4
DG04	4		√									1			√			√						√						3
DG05	10	√			√							2	√		√	√	√	√	√			√	√							8
DG06	4		√					√				2			√			√												2
DG07	6	√	√									2	√	√	√			√												4
DG08	6	√	√									2	√		√		√	√												4
DG09	7		√			√						2	√	√	√	√		√												5
DG10	10	√			√							2	√	√	√	√	√	√	√			√								8
DG11	4		√									1			√		√	√												3
DG12	10				√			√				2		√	√	√	√	√	√	√		√								8
DG13	7	√					√					2	√		√		√	√								√				5
DG14	6	√	√									2	√		√		√	√												4

DG15	8	√	√									2	√	√	√	√	√	√												6
DG16	7	√	√									2	√	√	√		√	√												5
DG17	8	√	√									2	√	√	√	√	√	√												6
DG18	5	√					√					2	√			√	√													3
DG19	4	√	√									2			√			√												2
DG20	4	√	√									2			√			√												2
DG21	6				√				√			2	√		√			√		√										4
DG22	7		√	√								2	√		√	√		√									√			5
DG23	6	√	√									2	√		√	√		√												4
DG24	7	√	√									2	√			√		√				√		√						5
DG25	6	√	√									2	√		√	√		√												4
DG26	5		√									1			√		√	√					√							4
DG27	7	√			√							2	√		√	√	√	√												5
DG28	8	√	√									2	√		√	√	√	√						√						6
DG29	5		√									1			√		√	√		√										4
DG30	5	√										1		√			√	√			√									4
DG31	6	√	√									2			√		√	√				√								4
DG32	9	√	√									2	√	√	√	√	√	√											√	7
DG33	5	√										1	√	√		√		√												4
DG34	5		√	√								2	√			√		√												3
DG35	5	√										1	√			√	√												√	4
DG36	5	√								√		2	√			√										√				3
DG37	6				√	√						2	√		√	√		√												4
DG38	4		√									1			√			√										√		3
DG39	5	√									√	2	√			√						√								3
DG40	8		√	√								2	√	√	√		√	√									√			6
DG41	5	√									√	2	√								√	√								3
DG42	8	√	√									2	√		√	√		√			√					√				6
DG43	6	√									√	2	√		√	√		√												4
DG44	6	√	√									2	√		√		√	√												4
DG45	5		√	√								2			√			√									√			3

DG46	4	√	√									2	√			√														2
DG47	6	√	√									2	√		√	√		√												4
DG48	4	√	√									2	√			√														2
DG49	7	√	√									2	√		√	√	√	√												5
DG50	5	√	√									2		√	√			√												3
DG51	3		√									1			√			√												2
DG52	5	√	√									2	√		√			√												3
DG53	3		√									1			√			√												2
DG54	4	√	√									2	√			√														2
DG55	6	√	√									2	√		√	√		√												4
DG56	6	√	√									2	√		√	√		√												4
DG57	4	√							√			1		√				√							√					3
DG58	3	√										1		√				√												2
DG59	5	√		√								2	√			√											√			3
DG60	3	√										1	√		√															2
DG61	6	√	√									2	√		√	√		√												4
DG62	5	√								√		2	√			√					√							√		3
DG63	4		√									1			√		√	√												3
DG64	3	√										1	√				√													2
DG65	6	√	√									2	√		√	√		√												4
DG66	3	√										1		√				√												2
DG67	6	√	√									2	√	√		√		√												4
DG68	5	√										1				√		√							√		√			4
	383	50	45	5	6	2	2	2	3	2	3		47	16	48	37	25	56	3	3	4	7	2	3	2	3	5	2	2	

"*01- *27"represents the allele Ancy-IE2*01-Ancy-IE2*27
