# Supplementary material for: Partial molecular characterization, expression pattern and polymorphism analysis of MHC I genes in Chinese domestic goose (Anser cygnoides)
Source: Genet Mol Biol. 2024 Jul 15;47(2):e20220252. doi: 10.1590/1678-4685-GMB-2022-0252 (PMC11249561; doi:10.1590/1678-4685-GMB-2022-0252)
Supplement: Table S4 - [file 1415-4757-GMB-47-02-e20220252-s4.pdf]

Genetics and Molecular Biology

DOI: https://doi.org/10.1590/1678-4685-GMB-2022-0252

Supplementary Material to “Partial molecular characterization, expression pattern and polymorphism analysis of MHC I genes in Chinese domestic goose (*Anser cygnoides*)”

Table S4 - Inference of positively selected amino acid sites for domestic goose MHC I sequences.

	PAML	FEL	MEMA
All	8V,17Q,42S,45N	8V, 45N	7E, 8V
Locus IA	8V	8V	8V
Locus non-IA	8V, 17Q, 42S, 45N	8V, 45N	7E, 8V

"42S" and "45N" correspond to the peptide-binding sites.
